# Cross-border introduction risk of Rift Valley fever virus: surveillance evidence from intercepted livestock in southwestern Saudi Arabia

**DOI:** 10.3389/fvets.2026.1893786

**Published:** 2026-08-19

**Authors:** Mohamed Siddig Alarabi, A. A. Altaher, Ommer M. Dafalla, Abdulla Ali Alashour, Fisal A. Bushlaibi, Fahad N. Abukhalil, Osama Ahmed Hassan, Abdelnassir Ahmed Taha

**Affiliations:** 1 WEQAA Center Laboratory in Jazan, The National Center for the Prevention and Control of Plants and Animal Diseases, Riyadh, Saudi Arabia; 2 Operation Sector, General Department of Laboratories, The National Center for the Prevention and Control of Plant Pests and Animal Diseases (Weqaa), Riyadh, Saudi Arabia; 3 Department of Clinical Microbiology, Faculty of Medicine, Umeå University, Umeå, Sweden; 4 Veterinary Section, Emirate Industry for Camel Milk and Milk Production, Dubai, United Arab Emirates

**Keywords:** One Health, Rift Valley fever, Saudi Arabia, seroprevalence, smuggled livestock, surveillance, transboundary disease, Yemen

## Abstract

**Introduction:**

Rift Valley fever (RVF) is a mosquito-borne transboundary zoonotic disease with major implications for animal and public health. Following the 2000 outbreak in Saudi Arabia and Yemen, the risk of reintroduction through cross-border livestock movement remains a continuing concern. This study aimed to estimate the seroprevalence of Rift Valley fever virus (RVFV) among illegally introduced livestock intercepted in the Jazan region of southwestern Saudi Arabia and to assess the added value of combined serological and molecular testing for detecting recent infection under routine surveillance conditions.

**Methods:**

Cross-sectional study was conducted using routine diagnostic data from confiscated livestock intercepted between January 2017 and December 2019.

**Results and discussion:**

A total of 2,202 serum samples from sheep, cattle, and camels were screened using a commercial competitive ELISA kit (ID.vet, France). Seroreactive samples were further characterized using IgM ELISA and real-time polymerase chain reaction (RT-PCR). Seroprevalence estimates were calculated with 95% confidence intervals (CI) and by species and year of collection. Agreement between IgM ELISA and RT-PCR was evaluated using Cohen’s kappa coefficient. Overall, 38 of 2,202 samples were cELISA-positive, giving an RVFV seroprevalence of 1.7% (95% CI: 1.3–2.4). Of the Seroreactive samples, 6/38 (15.8%; 95% CI: 7.4–30.4) were IgM-positive, indicating recent infection. Three samples were RT-PCR-confirmed, and all molecularly confirmed cases were also IgM-positive. Agreement between IgM and PCR was moderate (*κ* = 0.62). Seroprevalence was higher in sheep than cattle, but without statistical significance (OR = 1.61; 95% CI: 0.82–3.17; *p* = 0.190). These findings provide surveillance-based evidence of RVFV exposure and recent infection among livestock intercepted at a high-risk border interface. They support illegal cross-border livestock movement as a potential pathway for RVFV introduction, while not establishing the geographic source or transmission route. Strengthening integrated laboratory surveillance, border biosecurity, and One Health coordination is essential for early detection and risk reduction.

## Introduction

Rift Valley fever (RVF) is a mosquito-borne zoonotic disease of significant veterinary and public health importance, affecting a wide range of domestic animals and humans across Africa and the Arabian Peninsula (1, 2). The disease is caused by Rift Valley fever virus (RVFV), a Phlebovirus within the family Phenuiviridae, and is characterized in livestock by high abortion rates, neonatal mortality, and substantial economic losses (1, 3). In humans, infection is typically self-limiting but may progress to severe complications such as haemorrhagic fever, encephalitis, or ocular disease in a subset of cases (2, 4).

The epidemiology of RVF is complex and closely associated with environmental and ecological drivers, including rainfall patterns, flooding, and the proliferation of mosquito vectors, particularly species of Aedes and Culex (3, 5). These factors contribute to episodic outbreaks as well as low-level inter-epidemic virus circulation, which may persist undetected in endemic regions (5, 6). Increasing evidence suggests that climate variability and environmental changes may further influence the geographic expansion and transmission dynamics of RVFV (6, 7).

RVF is classified as a transboundary animal disease due to its capacity for rapid spread across regions through both vector dispersal and the movement of infected animals (7, 8). The first documented outbreak of RVF outside Africa occurred in 2000 in southwestern Saudi Arabia and neighboring Yemen, resulting in significant morbidity and mortality in both animals and humans (2, 9). Since then, the Jazan region has remained a high-risk area due to its ecological suitability for vector survival and its proximity to transboundary livestock movement routes (7, 9).

Unregulated or illegal livestock movement across the Saudi–Yemen border represents a critical but under-documented potential pathway for RVFV introduction. Such animals typically bypass veterinary inspection, quarantine procedures, and disease screening systems, thereby increasing the risk of introducing infected or incubating animals into susceptible populations (8, 10). Despite this plausible epidemiological pathway, there is limited field-based evidence supporting the role of intercepted livestock as a potential source of RVFV circulation in the region.

During the study period, the local surveillance included the interception of suspected illegally moved livestock by border-security authorities, followed by transfer to designated government quarantine facilities for veterinary inspection, diagnostic sample collection, and laboratory testing at the WEQAA Center Laboratory in Jazan. This risk-based, event-driven surveillance complemented routine animal-health monitoring in the region. As this study was based on retrospective surveillance records, shipment-level identifiers, interception totals, individual animal traceability data and movement history, were not consistently available and, therefore, could not be included in the analyses.

Previous investigations in Saudi Arabia have documented the 2000 RVF epizootic, subsequent intermittent virus detection in the affected southwestern area, and serological evidence of RVFV exposure in livestock across several regions of the country. However, studies specifically investigating RVFV in intercepted illegally moved livestock at the Saudi Arabia–Yemen border remain limited. The present study addresses this important gap by characterizing a high-risk border surveillance. Accordingly, the findings should be interpreted within the context of surveillance at the border interface and should not considered as evidence of the animals’ country of origin or the precise route of RVFV (11–13).

Surveillance systems that integrate serological and molecular diagnostic approaches are essential for detecting both historical exposure and active infection. Serological assays, such as ELISA, are widely used for population-level screening, while molecular methods such as real-time RT-PCR confirm active viraemia (4, 10). The combined use of these tools enhances the sensitivity and interpretability of surveillance data, particularly in high-risk border regions.

Therefore, this study aimed to estimate the seroprevalence of RVFV among illegally introduced livestock intercepted in the Jazan region of southwestern Saudi Arabia and to evaluate the added value of combining serological and molecular diagnostic methods for identifying recent and active infections under routine surveillance conditions. This highlights the increasing importance of integrated surveillance under changing climatic conditions.

## Materials and methods

### Study design and data source

A cross-sectional study was conducted using routine diagnostic data generated by the WEQAA Center Laboratory in Jazan, Saudi Arabia. The dataset included serum samples collected from illegally introduced livestock intercepted at border control points between January 2017 and December 2019. These animals were confiscated as part of routine veterinary surveillance activities targeting transboundary disease risks.

The study population comprised livestock intercepted during anti-smuggling operations rather than animal selected through probability-based field sampling. Following interception, animals were transferred to official holding facilities, where they underwent routine veterinary inspection and diagnostic sampling. All samples available in the laboratory database during the study period were included in the analysis. Because the retrospective surveillance records were incomplete, information on the total number of intercepted animals, shipment-level identifiers, age, sex, exact geographic origin, route of movement, or vaccination history was not consistently available.

### Study population

A total of 2,202 serum samples were included, comprising sheep (*n* = 1,185), cattle (*n* = 986), and camels (*n* = 31).

All samples were submitted to the laboratory under routine surveillance procedures, and no additional sampling was performed specifically for this study.

Sampling was operational and opportunistic, reflecting routine surveillance activities rather than a structured sampling design. Consequently, the analysed samples should not be considered representative of livestock populations in Yemen, Saudi Arabia, or any other source population; instead, they represent only intercepted animals from which diagnostic specimens were collected, and whose test results were available in the laboratory records.

## Serological testing

### Screening (IgG detection)

Initial screening for RVFV antibodies was performed using a commercial competitive enzyme-linked immunosorbent assay (cELISA) for the detection of RVFV-specific IgG antibodies (ID.vet, France), following the manufacturer’s instructions. This assay is widely used for surveillance purposes and allows detection of prior exposure to RVFV (4, 10).

### Detection of recent infection (IgM ELISA)

Samples that tested positive by cELISA were further analyzed using an IgM ELISA to identify recent infections. The presence of RVFV-specific IgM antibodies was interpreted as evidence of recent exposure, consistent with established diagnostic criteria (4, 5).

## Molecular detection (RT-PCR)

### RNA extraction

Viral RNA was extracted from serum samples using the MagNA Pure System (Roche Diagnostics, Germany), according to the manufacturer’s instructions. This automated extraction platform ensures standardized nucleic acid purification and minimizes contamination risks.

### RT-PCR assay

Detection of RVFV RNA was performed using a one-step real-time reverse transcription polymerase chain reaction (RT-PCR) assay in a ready-to-use monodose format, following standard protocols for RVFV detection. The assay targets conserved regions of the RVFV genome and is designed for rapid and sensitive detection of active viraemia (5, 10).

### Statistical analysis

Seroprevalence was calculated as the proportion of positive samples among the total tested population, with 95% confidence intervals (CI) estimated using the Wilson method. Comparisons between categorical variables (species and year) were performed using the chi-square test. Odds ratios (OR) with 95% CI were calculated to assess the strength of association between species and seropositivity.

Agreement between IgM ELISA and RT-PCR results was evaluated using Cohen’s kappa coefficient (*κ*), with interpretation based on standard thresholds (e.g., slight, fair, moderate, substantial agreement). All statistical analyses were performed using IBM SPSS Statistics for Windows, Version 26.0 (IBM Corp., Armonk, NY, USA). A *p*-value of < 0.05 was considered statistically significant.

A multivariable model adjusting for both year and species was considered. However, the archived dataset available for this retrospective analysis contained only aggregated data and lacked the individual-level cross-classification required for reliable multivariable regression. Constructing a multivariable model would therefore have required unverifiable assumptions and could have introduced bias. Accordingly, the analyses were restricted to univariable and descriptive approaches, and this limitation is acknowledged in the manuscript.

### Ethical considerations

The study was based on anonymized routine surveillance data collected as part of official veterinary activities. No additional animal handling or sampling was performed specifically for research purposes.

## Results

### Sample distribution

A total of 2,202 animal samples were collected during the study period from 2017 to 2019 (Table 1). Sheep constituted the highest proportion of samples overall (*n* = 1,185), followed by cattle (*n* = 986) and camels (*n* = 31).

**Table 1:** Samples distribution by species and year.

Year	Sheep	Cattle	Camels	Total
2017	337	294	1	632
2018	263	425	3	691
2019	585	267	27	879
Total	1,185	986	31	2,202

In 2017, a total of 632 samples were collected, with sheep accounting for the majority (*n* = 337), followed by cattle (*n* = 294) and camels (*n* = 1). The total number of samples increased in 2018 to 691, largely due to a rise in cattle samples (*n* = 425), while sheep and camel samples were 263 and 3, respectively. The highest number of samples was recorded in 2019 (*n* = 879), dominated by sheep (*n* = 585), with cattle and camel samples numbering 267 and 27, respectively.

Overall, sheep consistently represented the largest species sampled across all years, while camel samples remained comparatively low throughout the study period. The year 2019 showed a marked increase in total sample numbers compared with 2017 and 2018 (Table 1).

### Overall seroprevalence

Rift Valley fever virus (RVFV) antibodies were detected in 38 of 2,202 tested samples, giving an overall seroprevalence of 1.7% (95% CI: 1.3–2.4).

### Seroprevalence by species

Seroprevalence varied by animal species (Table 2). Sheep showed the highest proportion of seropositive samples (25/1,185; 2.1%), followed by cattle (13/986; 1.3%), while no RVFV antibodies were detected in camels (0/31; 0%). However, the difference in seroprevalence between species was not statistically significant (*p* = 0.281). The odds of seropositivity were higher in sheep compared with cattle (OR = 1.61), although this association was not statistically significant (*p* = 0.190).

**Table 2:** Seroprevalence of RVFV antibodies by species.

Species	Tested	Positive	%
Sheep	1,185	25	2.1
Cattle	986	13	1.3
Camels	31	0	0

### Seroprevalence by year

Annual seroprevalence estimates are presented in Table 3. RVFV antibodies were detected in 2.2% (14/632) of samples in 2017 and 2.3% (16/691) in 2018. In 2019, seroprevalence declined to 0.9% (8/879). Analysis across years suggested a borderline temporal trend in seroprevalence (*p* = 0.056).

**Table 3:** Seroprevalence of RVFV antibodies by year.

Year	Tested	Positive	%
2017	632	14	2.2
2018	691	16	2.3
2019	879	8	0.9

### IgM and PCR findings

The combined IgM serology and RT-PCR results are summarized in Table 4. IgM antibodies were detected in a subset of samples, indicating recent RVFV exposure, while only three samples were PCR-positive, suggesting limited active infection at the time of sampling. All PCR-positive samples were also IgM-positive. In contrast, three additional samples were IgM-positive but PCR-negative, consistent with post-viremic stages of infection. No PCR positivity was detected among IgM-negative samples.

**Table 4:** Combined IgM and PCR results for RVFV detection.

IgM	PCR+	PCR-
Positive	3	3
Negative	0	29

## Discussion

This study provides surveillance-based evidence of low-level RVFV exposure and recent infection among illegally introduced livestock intercepted in the Jazan region of southwestern Saudi Arabia. These findings are epidemiologically important because they were generated from a high-risk border surveillance population. However, they should not be interpreted as evidence of animals’ geographic origin, local vector-borne transmission, or proof that illegal livestock movement resulted in RVFV reintroduction into the region.

The overall seroprevalence of 1.7% was lower than estimates reported in livestock serosurveys conducted in endemic regions of Africa, where seroprevalence varies considerably according to ecological condition, host species, outbreak history, and sampling design. In Saudi Arabia, previous studies have documented the 2000 RVF outbreak, subsequent intermittent RVFV activity in the southwestern region, and serological evidence of exposure in livestock, supporting the need for continued surveillance and periodic seromonitoring (11, 12). Direct comparison with previous studies should be interpreted cautiously because the present study was based on opportunistic surveillance of intercepted livestock rather than probability-based sampling. Consequently, the study population differs from those included in structured regional or her-based serosurveys, limiting the comparability of the prevalence estimates.

The presence of IgM antibodies in a subset of seropositive animals (15.8%) highlights recent exposure to RVFV, while the detection of viral RNA in a smaller proportion of samples confirms active viraemia at the time of sampling. This combination of findings is consistent with inter-epidemic transmission patterns described in endemic regions, where low-level virus circulation persists between major outbreaks (5, 6). Importantly, even limited detection of recent infection should not be underestimated, particularly in ecologically suitable regions where vector populations can rapidly amplify transmission under favorable environmental conditions (6, 7).

Illegal livestock movement represents a critical epidemiological pathway for transboundary disease introduction. Animals entering through informal routes bypass established veterinary inspection systems, including quarantine and health certification, thereby increasing the risk of introducing infected or incubating animals into susceptible populations (8, 9). The detection of RVFV exposure among intercepted animals in this study provides empirical support for this pathway and underscores the importance of strengthening border surveillance systems.

The observed difference in seroprevalence between sheep and cattle, although not statistically significant, is consistent with previous studies indicating higher susceptibility of small ruminants to RVFV infection and their potential role in virus amplification (1, 3). The absence of seropositive cases in camels should be interpreted with caution due to the limited sample size, which restricts meaningful epidemiological conclusions for this species.

Temporal variation in seroprevalence, with higher rates observed in 2017 and 2018 compared to 2019, may reflect fluctuations in environmental conditions such as rainfall and vector abundance. These findings are in line with previous studies demonstrating the influence of climatic factors on RVFV transmission dynamics (6, 7). Although the observed trend approached statistical significance, it did not meet the conventional threshold, suggesting that longer-term data would be required to confirm temporal patterns.

The moderate agreement (*κ* = 0.62) between IgM ELISA and RT-PCR reflects the biological differences in detection windows between serological and molecular methods. While RT-PCR is limited to detecting viral RNA during the viraemic phase, IgM antibodies may persist beyond the period of detectable viraemia (4, 10). The absence of PCR-positive results among IgM-negative samples supports the reliability of molecular detection, while IgM-positive/PCR-negative cases likely represent animals in the early convalescent or post-viraemic stage. These findings highlight the complementary value of combining serological and molecular tools in surveillance systems.

Diagnostic uncertainty should also be considered when interpreting the findings. The likelihood of false-positive and false-negative results depends on assay sensitivity, specificity, specimen quality, and the timing of sample collection relative to infection. RT-PCR is most sensitive during the relatively short viraemic phase, whereas IgM may remain detectable after viral RNA has declined below the assay detection limit. Accordingly, IgM-positive/RT-PCR-negative results are consistent with recent infection during the post-viraemic or early convalescent phase, although diagnostic misclassification cannot be excluded. Likewise, a negative PCR result does not rule out recent infection if sampling occurred outside the viraemic window. Confirmation by virus neutralization testing, repeat sampling, virus isolation, or genome sequencing would have increased diagnostic certainty (10, 14–16).

From a surveillance perspective, intercepted livestock represent a valuable early warning system for transboundary animal diseases. The integration of serological screening with confirmatory molecular diagnostics enhances the ability to detect both historical exposure and active infection, thereby improving the sensitivity and interpretability of surveillance data. In high-risk border regions such as Jazan, such integrated approaches are essential for timely detection and response.

Overall, these findings indicate that intercepted livestock constitute a valuable sentinel population for border surveillance and are consistent with illegal cross-border animal movement representing a potential pathway for RVFV introduction. However, the results do not permit causal inference or source attribution. Addressing these questions will require additional evidence, including shipment-level epidemiological data, contemporaneous vector surveillance, and viral genomic sequencing with phylogenetic analysis.

Strengthening surveillance through an integrated One Health approach would improve early detection and interpretation of RVFV activity by linking animal health surveillance with human case surveillance, entomological monitoring, environmental and climatic indicators, livestock movement intelligence, and genomic data. Such integration is particularly important for RVF because virus transmission is shaped by the interaction of susceptible animal hosts, competent mosquitoes’ vectors, environmental conditions and human exposure (17, 18).

These findings have important implications for regional disease control policies and cross-border surveillance strategies.

## Conclusion

This study detected RVFV specific antibodies, IgM antibodies, and viral RNA in illegally introduced livestock intercepted in the Jazan region of southwestern Saudi Arabia, providing surveillance-based evidence of previous exposure and recent infection within a high-risk border surveillance population.

These findings highlight the value of intercepted livestock as a sentinel population for border surveillance and support continued risk-based monitoring at high-risk border interfaces.

Future efforts should focus on strengthening border biosecurity and implementing integrated One Health surveillance to enable timely detection, risk assessment, and coordinated response to RVFV activity. Complementary epidemiological investigations, vector surveillance, shipment traceability, and viral genomic characterization will be important for clarifying routes of virus introduction and informing prevention and control strategies.

### Limitations

This study has several limitations that should be considered when interpreting the findings. First, the retrospective design relied on routine surveillance data, which may limit the completeness and consistency of available information. Second, detailed data on the geographic origin, movement history, and vaccination status of the animals were not available, restricting the ability to fully assess epidemiological links and risk factors.

Third, the absence of entomological (vector) surveillance data prevented correlation of serological findings with vector abundance and environmental conditions. Additionally, the relatively small number of camel samples limits the interpretation of species-specific findings for this group.

Finally, the data were collected between 2017 and 2019 and therefore may not fully reflect the current epidemiological situation. However, the study provides valuable insight into inter-epidemic RVFV circulation and highlights the importance of surveillance in high-risk border regions.

Additional limitations include the absence of confirmatory virus-neutralisation testing, repeat sampling, and viral genomic sequencing with phylogenetic analysis of the RT-PCR-positive samples. Consequently, the viral lineage, geographic source, and route of introduction could not be determined. Furthermore, the lack of individual-level, cross-classified data precluded reliable multivariable analysis adjusting simultaneously for species and year.

## Data Availability

The raw data supporting the conclusions of this article will be made available by the authors, without undue reservation.

## References

[1] Mansfield KL Banyard AC McElhinney LM Johnson N Horton DL Hernández-Triana LM . Rift Valley fever virus: a review of diagnosis and vaccination, and implications for emergence in Europe. Vaccine. (2015) 33:5520–31. doi: 10.1016/j.vaccine.2015.08.020, 26296499

[2] Shoemaker T Boulianne C Vincent MJ Pezzanite L Al-Qahtani MM Al Mazrou Y . Genetic analysis of viruses associated with emergence of Rift Valley fever in Saudi Arabia and Yemen, 2000–01. Emerg Infect Dis. (2002) 8:1415–20. doi: 10.3201/eid0812.020195 12498657 PMC2738516

[3] Madani TA Al-Mazrou YY Al-Jeffri MH Mishkhas AA Al-Rabeah AM Turkistani AM . Rift Valley fever epidemic in Saudi Arabia: epidemiological, clinical, and laboratory characteristics. Clin Infect Dis. (2003) 37:1084–92. doi: 10.1086/378747, 14523773

[4] Davies FG Martin V. Recognizing Rift Valley fever. Rome: Food and Agriculture Organization; (2003).

[5] Mohamed M Mosha F Mghamba J Zaki SR Shieh WJ Paweska J . Epidemiologic and clinical aspects of a Rift Valley fever outbreak in humans in Tanzania, 2007. Am J Trop Med Hyg. (2007) 83:22–7. doi: 10.4269/ajtmh.2010.09-0318, 20682902 PMC2913502

[6] Lambin EF Tran A Vanwambeke SO Linard C Soti V. Pathogenic landscapes: interactions between land, people, disease vectors, and their animal hosts. Int J Health Geogr. (2010) 9:54. doi: 10.1186/1476-072X-9-54, 20979609 PMC2984574

[7] Lancelot R Béral M Rakotoharinome VM Andriamandimby SF Héraud JM Coste C . Rift Valley fever: recent insights into pathogenesis and epidemiology. Proc Natl Acad Sci USA. (2017) 114:938–43. doi: 10.1073/pnas.1607948114 28096420 PMC5293023

[8] Jupp PG Kemp A Grobbelaar A Leman P Burt FJ Alahmed AM . The 2000 epidemic of Rift Valley fever in Saudi Arabia: mosquito vector studies. Med Vet Entomol. (2002) 16:245–52. doi: 10.1046/j.1365-2915.2002.00371.x, 12243225

[9] Balkhy HH Memish ZA. Rift Valley fever: an uninvited zoonosis in the Arabian peninsula. Int J Antimicrob Agents. (2003) 21:153–7. doi: 10.1016/S0924-8579(02)00295-9, 12615379

[10] Wilson WC Romito M Jasperson DC Weingartl H Binepal YS Maluleke MR . Development of a Rift Valley fever real-time RT-PCR assay that can detect all three genome segments. J Virol Methods. (2013) 193:426–431. doi: 10.1016/j.jviromet.2013.07.006 23850696

[11] Al-Afaleq AI Hussein MF. The status of Rift Valley fever in animals in Saudi Arabia: a mini review. Vector Borne Zoonotic Dis. (2011) 11:1513–20. doi: 10.1089/vbz.2010.0245, 21923257

[12] Al-Afaleq AI Hussein MF Al-Naeem AA Housawi F Kabati AG. Seroepidemiological study of Rift Valley fever in animals in Saudi Arabia. Trop Anim Health Prod. (2012) 44:1535–39. doi: 10.1007/s11250-012-0100-x 22359088

[13] Hassan OA Ahlm C Sang R Evander M. The 2007 Rift Valley fever outbreak in Sudan. PLoS Negl Trop Dis. (2011) 5:e1229 21980543 10.1371/journal.pntd.0001229 PMC3181235

[14] Drosten C Göttig S Schilling S Asper M Panning M Schmitz H . Rapid detection and quantification of RNA of Ebola and Marburg viruses, Lassa virus, Crimean-Congo haemorrhagic fever virus, Rift Valley fever virus, dengue virus, and yellow fever virus by real-time reverse transcription-PCR. J Clin Microbiol. (2002) 40:2323–30. doi: 10.1128/JCM.40.7.2323-2330.2002, 12089242 PMC120575

[15] Sall AA Macondo EA Sène OK Diagne M Sylla R Mondo M . Use of reverse transcriptase PCR in early diagnosis of Rift Valley fever. Clin Diagn Lab Immunol. (2002) 9:713–5. doi: 10.1128/CDLI.9.3.713-715.2002, 11986283 PMC119982

[16] World Organisation for Animal Health (WOAH). "Infection with Rift Valley fever virus". In: Manual of Diagnostic Tests and Vaccines for Terrestrial Animals. Paris: WOAH (2023)

[17] Fawzy M Helmy YA. The one health approach is necessary for the control of Rift Valley fever infections in Egypt: a comprehensive review. Viruses. (2019) 11:139. doi: 10.3390/v11020139, 30736362 PMC6410127

[18] Food and Agriculture Organization of the United Nations. Rift Valley fever Surveillance. Rome: FAO (2018).

